# Jejunal volvulus within an inguinal hernia sac like as an extremely rare cause of acute mechanical gastrointestinal obstruction in adults: First literature report

**DOI:** 10.1016/j.ijscr.2022.106757

**Published:** 2022-01-28

**Authors:** Giuseppe Evola, Mario Scravaglieri, Giovanni Francesco Di Fede, Carla Di Stefano, Salvatore Sarvà, Luigi Piazza

**Affiliations:** a General and Emergency Surgery Department, Garibaldi Hospital, Piazza Santa Maria di Gesù 5, 95100 Catania, Italy; b Department of Radiology, Santa Marta e Santa Venera Hospital, Via Caronia, 95024 Acireale (Catania), Italy

**Keywords:** Small bowel volvulus, Gastrointestinal obstruction, Abdominal pain, Emergency surgery, Case report

## Abstract

**Introduction and importance:**

Small bowel volvulus (SBV) represents a rare and life-threatening cause of gastrointestinal obstruction among adults. SBV can be classified as primary and secondary subtypes. Preoperative diagnosis of SBV is a challenge because of the absence of pathognomonic clinical, radiographic and laboratory findings. Surgery represents the correct treatment of SBV.

**Case presentation:**

A 69-year-old Caucasian male presented to the Emergency Department with a two-day history of abdominal pain, inability to pass gas or stool, nausea, vomiting. Physical examination revealed abdominal distension, generalized abdominal pain without guarding or rebound tenderness, a partially reducible and painless right inguinal hernia. Laboratory tests reported neutrophilic leukocytosis. Abdominal computed tomography revealed massive gastroduodenal dilatation with pneumoperitoneum and small bowel loops in the right inguinal sac. The patient underwent exploratory laparotomy: a jejunal volvulus (JV) located within the right inguinal hernia sac, causing gastrointestinal obstruction, was devolvulated and a right prosthetic inguinal hernia repair was also performed. The patient was discharged on the 10th postoperative day.

**Clinical discussion:**

Secondary SBV is due to any congenital or acquired lesions and rarely occurs among adults in Western countries. This is the first literature report of a JV located within an inguinal hernia sac causing gastrointestinal obstruction.

**Conclusion:**

Secondary JV represents an extremely rare abdominal emergency necessitating early diagnosis to prevent the development of intestinal ischemia, bowel necrosis and peritonitis. Diagnosis of JV needs a high index of suspicion and may be facilitated by imaging, often it is made intraoperatively. Surgery represents the appropriate treatment of JV.

## Highlights

• Jejunal volvulus (JV) represents a rare cause of gastrointestinal obstruction among adults.
• Diagnosis is a challenge because of the absence of specific clinical presentation, pathognomonic radiographic and laboratory findings.
• Abdominal contrast-enhanced computed tomography (CECT) remains the most relevant imaging modality for diagnosis.
• Early diagnosis and early surgical intervention are the keys for the successful management of JV.

## 1 Introduction

Small bowel volvulus (SBV), characterized by torsion of a segment of small bowel and its mesentery, represents a rare and life-threatening cause of gastrointestinal obstruction, accounting 1–4% cases in Western World but up to 20–35% cases in Asia, Africa and Middle East [1]. SBV can be classified as primary, without any predisposing factors, or secondary to any congenital or acquired lesions. Preoperative diagnosis of SBV is very difficult because of the absence of specific clinical presentation and pathognomonic radiographic and laboratory findings [2]. Abdominal contrast-enhanced computed tomography (CECT) remains the most relevant imaging modality for diagnosis. An extremely rare case of secondary jejunal volvulus (JV) located within an inguinal hernia sac, reported for the first time in the literature, is presented in accordance with SCARE 2020 criteria [3]. The purpose of this case report is to remember that JV represents an extremely rare cause of acute abdomen that requires emergency surgery.

## 2 Presentation of case

**Fig. 1:**
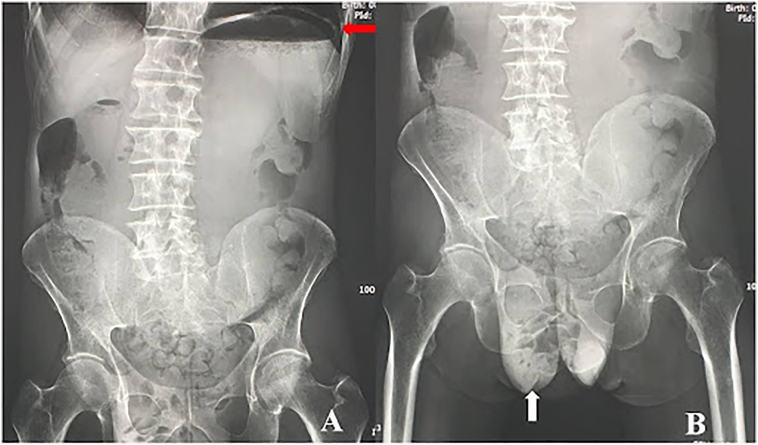
A,B. Abdominal radiography showing gastric dilatation with suspected pneumoperitoneum (Fig. 1 A, red arrow) and intestinal loops in the right inguinal hernia sac (Fig. 1 B, white arrow). (For interpretation of the references to colour in this figure legend, the reader is referred to the web version of this article.)

**Fig. 2:**
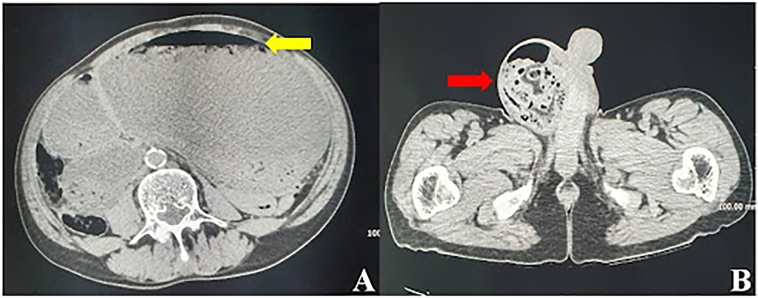
A,B. Preoperative abdominal computed tomography scan (transverse view) revealing massive gastroduodenal dilatation with pneumoperitoneum (Fig. 2 A, yellow arrow) and small bowel loops in the right inguinal hernia sac (Fig. 2 B, red arrow). (For interpretation of the references to colour in this figure legend, the reader is referred to the web version of this article.)

**Fig. 3:**
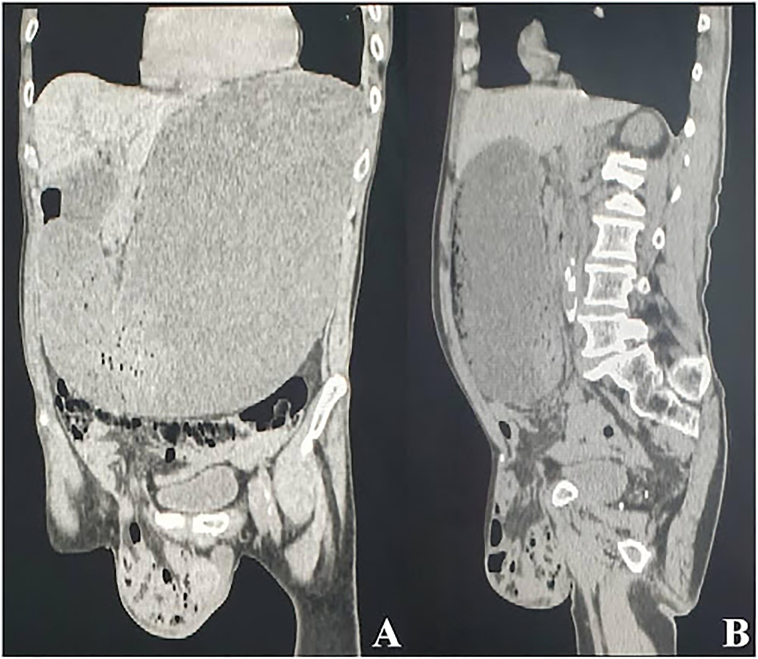
A,B. Preoperative abdominal computed tomography scan revealing massive gastroduodenal dilatation and small bowel loops in the right inguinal hernia sac. Fig. 3 A coronal view, Fig. 3 B sagittal view.

**Fig. 4:**
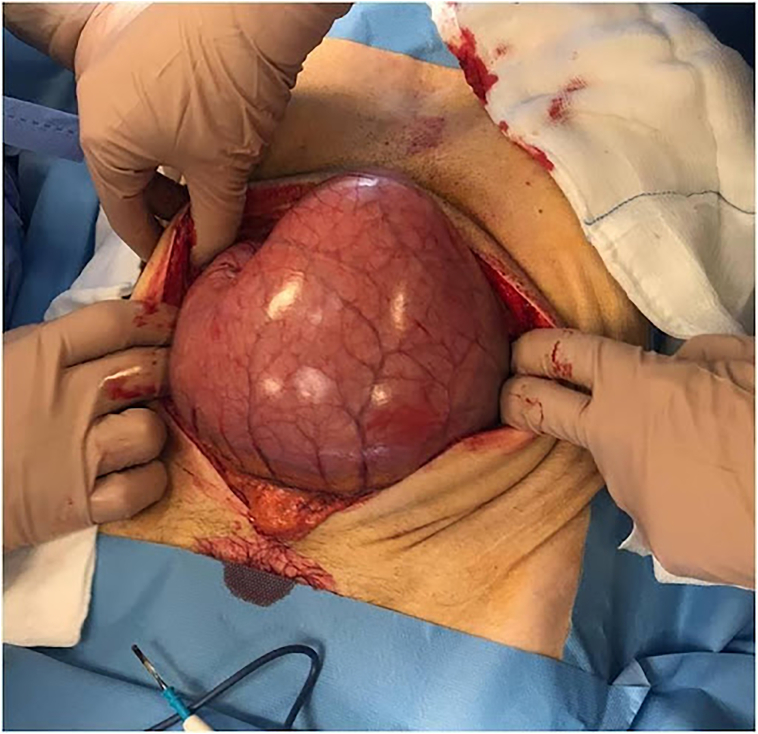
Massive dilatation of the stomach.

**Fig. 5:**
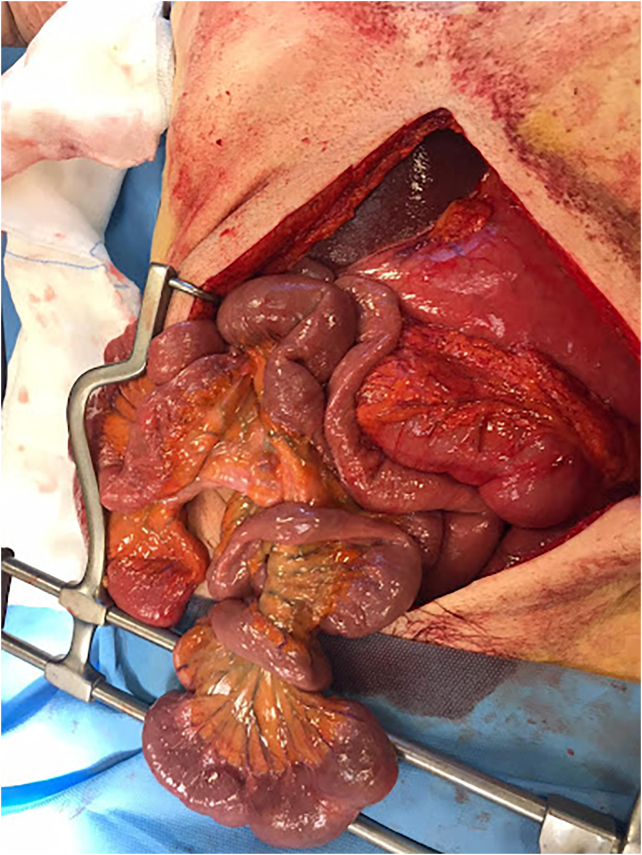
Jejunal volvulus located within the right inguinal hernia sac.

A 69-year-old Caucasian male presented to the Emergency Department with a two-day history of abdominal pain, inability to pass gas or stool, nausea, vomiting; vital signs were normal. He referred habit on smoking and alcohol consumption; there was no history of previous abdominal surgery or changes in dietary habits. His past and familial medical histories were normal. The patient was retired from the work, married and of medium socio-economic status. Physical examination revealed abdominal distension, severe and generalized abdominal pain without guarding or rebound tenderness, hypoactive bowel sounds and a partially reducible and painless right inguinal hernia. Laboratory tests reported neutrophilic leukocytosis (WBC 13.700 10^3^/μL). The patient was initially managed with fluids, intravenous broad-spectrum antibiotics and bowel rest. After abdominal radiography showing gastric dilatation with suspected pneumoperitoneum and intestinal loops in the right inguinal hernia sac (Fig. 1), the patient was evaluated by abdominal computed tomography (CT) scan which revealed massive gastroduodenal dilatation with pneumoperitoneum and small bowel loops in the right inguinal hernia sac (Figs. 2, 3). The patient, after understanding the severity of his medical condition and accepting surgery, was taken emergently to the operating room by experienced general surgeons for exploratory laparotomy under general anesthesia. The patient was placed in the supine position on the operating table: intraoperatively we found a massive dilatation of the stomach (Fig. 4), duodenum and proximal small bowel loops caused by a JV located within the right inguinal hernia sac (Fig. 5). There was 720° anticlockwise rotation of a 30 cm segment of the jejunum around its mesentery forming a volvulus 80 cm distal to the ligament of Treitz; intestinal loops distal to the obstruction were collapsed. Diagnosis of acute mechanical gastrointestinal obstruction, caused by JV, was made intraoperatively. Gastroduodenal dilatation was detained by a nasogastric tube, JV was devolvulated rotating the segment in clockwise direction. No signs of ischemia or vascular compromise were found so small bowel resection wasn't necessary and a pelvic drain was placed. Methylene blue test and air test through the nasogastric tube excluded gastroduodenal and jejunal perforation. Right prosthetic inguinal hernia repair was also performed. Patient was given an IV injection of Amoxicillin/Clavulanate 2 g twice daily and Metronidazole 500 mg thrice daily for five days and a SC injection of enoxaparin sodium 4.000 IU once daily for 21 days. Prolonged ileus for five days which resolved spontaneously was followed by an uneventful recovery; laboratory tests were unremarkable. The patient was discharged on the 10th postoperative day, after removal of abdominal drain, in a stable condition; he tolered the advice provided and after a follow-up of six months is asymptomatic.

## 3 Discussion

This clinical case describes an extremely rare JV causing gastrointestinal obstruction. Volvulus occurs most commonly in the large bowel than in the small bowel and stomach [4]. SBV is a rare condition among adults where the bowel loops coiled around the axis of its own mesentery. A twist of small bowel loops greater than 180° around its mesenteric vascular pedicle results in acute mechanical gastrointestinal obstruction and in vascular inflow and outflow compromise, leading to bowel ischemia and necrosis, bowel perforation and peritonitis [5]. SBV can be classified as primary and secondary subtypes according to the cause. Primary SBV occurs without underlying anatomical abnormalities or predisposing factors and it is observed mainly in children and young adults; it is more common in Africa and Asia continent. Many mechanisms of primary SBV have been suggested including a strong anterior abdominal muscle tone, high peristaltic tone of the bowel, a bulky higher fiber meal in the small bowel after a prolonged period of fasting, longer mesenteric length and shortness of the mesenteric root allowing abnormal mobility of a small bowel segment [6]. Secondary SBV is usually found between the age of 40 and 90 years [6], it is more common in Western countries and makes up 78% to 90% of SBV [7]. Secondary SBV is mainly due to postoperative adhesions, fibrous band, Meckel's diverticulum, congenital malrotation of the gut, tumours, mesenteric lymph nodes, parasitic infestations, internal hernias, lipomas, pregnancy, endometriosis, hematomas, aneurysms, tuberculosis, intestinal duplication, jejunal diverticulum, small bowel diverticula, paraduodenal hernia. [6], [8], [9]. This is the first case of a JV secondary to a inguinal hernia reported in the literature: we suppose that the presence of jejunal loops within the inguinal hernia sac have induced forceful bowel peristalsis resulting in JV. Diagnosis of SBV is difficult due to its rarity and nonspecific clinical presentation. A high index of suspicion is required and an early diagnosis is essential to avoid mesenteric ischemia and bowel necrosis. SBV may present either acutely (89%) due to acute vascular insufficiency or peritonitis, or else with vague symptoms and signs (abdominal pain, nausea, vomiting, abdominal distension, a decrease in flatus production) [10] that are common to others causes of gastrointestinal obstruction [11], [12], [13]. In our case report the patient presented symptoms and signs of gastrointestinal obstruction without peritoneal signs. No laboratory findings of SBV are specific as in our case. Preoperative diagnostic workup of SBV includes plain abdominal radiography, ultrasonography (US), Color Doppler US and abdominal CECT [5]. Plain abdominal radiography has low accuracy in diagnosing SBV, it can demonstrate nonspecifically signs of intestinal obstruction (air-fluid levels, dilated bowel loops), ischemia or necrosis (thumbprinting, pneumatosis intestinalis, portal vein gas). US is operator dependent and can show the twisting of small bowel around its mesentery [14]. Color Doppler US can demonstrate the encircling of the small bowel loops and the superior mesenteric vein around the superior mesenteric artery, which is termed the “whirlpool sign”, with a sensitivity, specificity and positive predictive value of 92%, 100% and 100% respectively [15]. Abdominal CECT represents the investigation of choice with a sensitivity of 60%–100% and a specificity of 90%–95% [16]: it can demonstrate “whirl sign”, “spoke wheel sign”, “beak sign”, “barber pole signs”, signs of small bowel obstruction (dilatation of closed or air-filled bowel loops) and ischemia (thickening or presence of air in the bowel wall, portal vein gas, free peritoneal fluid); however none of these findings is pathognomonic of SBV [8]. M. Lepage-Saucier et al. observed on abdominal CECT three signs of SBV which are multiple transition points, transition points located ≤7 cm from the spine in the anteroposterior plane and the whirl sign: the presence of any one of these signs confirms SBV with a sensitivity of 94%, the presence of all signs confirms SBV with100% specificity [1]. In our case report abdominal radiography showed gastric dilatation with suspected pneumoperitoneum and intestinal loops in the right inguinal sac, abdominal CT scan revealed massive gastroduodenal dilatation with pneumoperitoneum and small bowel loops in the right inguinal sac and diagnosis of JV was made intraoperatively. The management of SBV is strictly surgical, conservative management is not indicated because the twisted intestinal loops carry an high risk of gangrene and peritonitis. Emergency surgery is the correct treatment to be undertaken with the aim of untwisting the SBV and re-establishing the intestinal blood flow. If small bowel is necrotic, resection and primary intestinal anastomosis or stoma are necessary [16]. The incidence of gangrenous bowel requiring resection has been reported as 15–50% [17]. Although it is clear that secondary SBV causes must be corrected during surgery, there is a continuing controversy regarding the surgical management of primary SBV: some authors considered the simple devolvulation as the most appropriate operation, others recommend additional intestinal fixation or even suggest resection to avoid recurrence (up to 30% of cases) [18]. In our case report only devolvulation of JV and a right prosthetic inguinal hernia repair were performed. Delay in diagnosis and surgery increases morbidity and mortality rates. Mortality rates range between 9% and 35% [19], but with gangrenous bowel mortality rates as high as 20–100% have been reported [17], [20].

## 4 Conclusion

JV represents an extremely rare surgical emergency that should be considered in the differential diagnosis of patients with acute gastrointestinal obstruction. Its diagnosis is a challenge because of the absence of pathognomonic clinical, radiographic and laboratory findings and needs a high index of suspicion. Early diagnosis and early surgical intervention are the keys for the successful management of JV.

## Sources of funding

This research did not receive any specific grant from funding agencies in the public, commercial, or not-for-profit sectors.

## Ethical approval

Ethical approval has been exempted by our institution because this is a case report and no new studies or new techniques were carried out.

## Consent

Written informed consent was obtained from the patient, for publication of this case report and accompanying images. A copy of the written consent is available for review by the Editor-in-Chief of this journal on request.

## Author's contribution

Giuseppe Evola: Operated on the patient, drafting the manuscript, literature research.

Mario Scravaglieri: Operated on the patient, drafting the manuscript.

Giovanni Francesco Di Fede: Drafting the manuscript, literature research.

Carla Di Stefano: Drafting the manuscript, literature research.

Salvatore Sarvà: Drafting the manuscript and literature research.

Luigi Piazza: Revising the manuscript.

## Registration of research studies

Not applicable.

## Guarantor

Giuseppe Evola.

## Provenance and peer review

Not commissioned, externally peer-reviewed.

## Declaration of competing interest

The authors have no conflict of interest to declare.
